# Prevention of lymphoedema after axillary clearance by external compression sleeves PLACE randomised trial results. Effects of high BMI

**DOI:** 10.1002/cam4.5378

**Published:** 2022-12-12

**Authors:** Nigel J. Bundred, Emma Barrett, Chriss Todd, Julie Morris, Donna Watterson, Arnie Purushotham, Katie Riches, Abigail Evans, Anthony Skene, Vaughan Keeley, the Investigators of BEA/PLACE studies

**Affiliations:** ^1^ Manchester University NHS Foundation Trust Manchester UK; ^2^ University of Manchester, Manchester Academic Health Sciences Centre (MAHSC) Manchester Manchester UK; ^3^ School of Health Sciences, Faculty of Biology, Medicine and Health The University of Manchester Manchester UK; ^4^ King's College London London UK; ^5^ University Hospitals of Derby and Burton NHS Foundation Trust Derby UK; ^6^ Poole NHS Foundation Trust Poole UK; ^7^ Royal Bournemouth NHS Foundation Trust Bournemouth UK; ^8^ University of Nottingham Medical School Nottingham UK

**Keywords:** axillary node clearance, BMI, breast cancer, compression sleeves, lymphoedema

## Abstract

Around 25% of women undergoing Axillary Clearance (ANC) develop lymphedema (LE). Intervention with a compression garment is recommended to prevent LE but no randomised evidence exists to support this strategy.

**Methods:**

A randomised trial tested standard management versus application of graduated compression garments (20‐24 mmHg) to affected arm, for 1 year. Women with node positive breast cancer (*n* = 1300) undergoing ANC consented to arm volume measurements and those developing a 4–9% relative arm volume increase (RAVI) (subclinical LE) within 9 months post‐surgery were randomised. Primary outcome was proportion of patients developing LE (RAVI > 10%) by 24‐months in each group. Secondary endpoints included Quality of life in each group.

**Results:**

In total 143 patients were randomised (74 no sleeve: 69 compression sleeve) between October 2010 and November 2015. The lymphoedema rate at 24 months in the ‘no sleeve’ group was at 41%, similar to the ‘sleeve’ group (30%: *p* = 0.32). Thirtytwo patients randomised to the ‘no sleeve’ group had a sleeve applied within 24 months. Body Mass Index (BMI) at randomisation predicted LE at any time point HR 1.04 (CI 1.01–1.08; *p* = 0.01). Patients with obesity (BMI > 30) had higher rates of LE in both groups (46%) compared to those with BMI < 30 (24%). No difference between patients was found in either group in changes in QoL. Compression sleeves applied after development of LE improved QoL scores (FACT‐B *p* = 0.007:TOI *p* = 0.042).

**Conclusion:**

Early intervention with External Compression garments does not prevent clinical LE, particularly in women with a high BMI > 30. The use of prophylactic garments in subclinical LE (RAVI < 9%) is unwarranted.

## Graphical abstract

A Randomised trial of external arm compression garments after axillary node clearance in women developing early arm swelling failed to find an effect of the intervention in preventing lymphoedema.

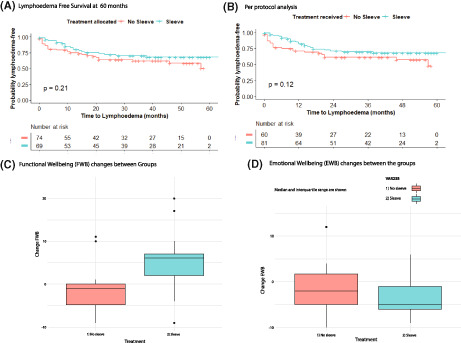

## 1 INTRODUCTION

As survival for breast cancer has improved with better treatments, management of the long‐ term complications that reduce patient's quality of life (QoL) is increasingly important for patient care.^1^, ^2^, ^3^, ^4^, ^5^ Breast cancer related arm lymphoedema (LE) is swelling of the arm after surgery or radiotherapy to the axilla.^1^, ^3^, ^5^, ^6^, ^7^, ^8^ LE is a progressive condition with initial fluid accumulating in the interstitial space of the subcutaneous tissue, followed by chronic inflammation, which leads to fibrotic thickening of skin and dermis.^7^ LE causes physical and psychosocial morbidity, with altered body image and recurrent infections of the arm (cellulitis) leading to progression of lymphoedema by further damage to lymph vessels.^3^, ^5^, ^7^

Increases in the relative ipsilateral arm volume (versus the contralateral arm) of more than 10% is accepted criteria for the diagnosis of lymphoedema.^3^, ^5^, ^7^, ^8^, ^9^ Most patients develop LE within 12–24 months of surgery and it is claimed early intervention after surgery may benefit patients.^3^, ^5^, ^6^, ^7^

Both a meta‐analysis and a large prospective UK study found high Body Mass Index (BMI) predicted development of lymphedema after axillary node clearance (dissection) surgery.^6^, ^7^ High BMI after development of LE also predicted earlier progression of lymphoedema.^7^

It is unknown whether BMI interferes with the ability of garment sleeves to provide sufficient compression to the arm.

A small US army cohort study of 43 women (in a group of patients with early 3–9% RAVI arm swelling) treated with arm sleeves for a median 4.4 months claimed early intervention of the compression arm sleeves prevented the development of chronic lymphoedema.^10^ This study asserted that early intervention prevented further arm swelling and QoL improved.^10^ Nearly all the patients in this study had a normal BMI.

Both in the United States in the National Lymphoedema Network Guidelines^11^ and in the International Lymphoedema Framework guidelines,^9^ surveillance strategies to identify patients developing lymphoedema after surgery have been introduced based on the Stout‐Giegich data, compared to the paradigm that addressed LE once it had developed.

Compression garments, which reduce the amount of interstitial fluid, are graduated with the greatest compression at the distal end and the least compression at the proximal end thus covering the entire area of oedema.^1^, ^9^, ^11^ The evidence base for these treatments in established LE is poor quality, with only three single centre randomised studies involving 150 patients with lymphoedema, none of which involved the same interventions.^12^ Reductions in arm swelling of 4–24% were found in studies of established lymphoedema.^12^

Early arm swelling predicts development of lymphoedema.^7^ Arm swelling of 4–9% is not usually clinically apparent unless arm measurements have been made pre‐operatively, but has been shown to predict an increased risk of lymphoedema.^7^, ^10^, ^11^

A trial of Manual Lymphatic Drainage (MLD) compared to arm exercises in women with early arm swelling after axillary surgery found that 28% control and 24% MLD patients developed LE (defined as Relative Arm Volume Increase (RAVI) > 10%) and concluded manual lymphatic drainage did not prevent LE development.^13^

Currently there is no large randomised trial evidence to support the value of compression garments in preventing lymphoedema after ANC. We tested the hypothesis whether early intervention in a group of patients with increased lymphoedema risk (RAVI 4–9% arm swelling) using a compression garment and supportive treatment compared to supportive treatment alone (written advice, arm elevation exercises and massage) reduces the subsequent development of lymphoedema and improves QoL in patients presenting through a surveillance programme following surgery who had developed a RAVI of 4–9%.

## 2 METHODS

As part of another prospective study (BEA)^7^ which compared bioimpedance spectroscopy with limb volume measurements in the early detection of LE after breast cancer treatment, women scheduled to undergo ANC gave consent to have baseline and follow‐up arm volume measurements by Perometer at 1, 3, 6 and 9 months after surgery in nine UK centres (http://isrctn.com/ISRCTN48880939). Perometer (http://www.pero‐system.de/wirueber_e.htm) arm volume measurements are reproducible, validated and have a low inter‐test variation in both normal volume human arms and lymphoedema arms.^7^, ^14^, ^15^

**FIGURE 1:**
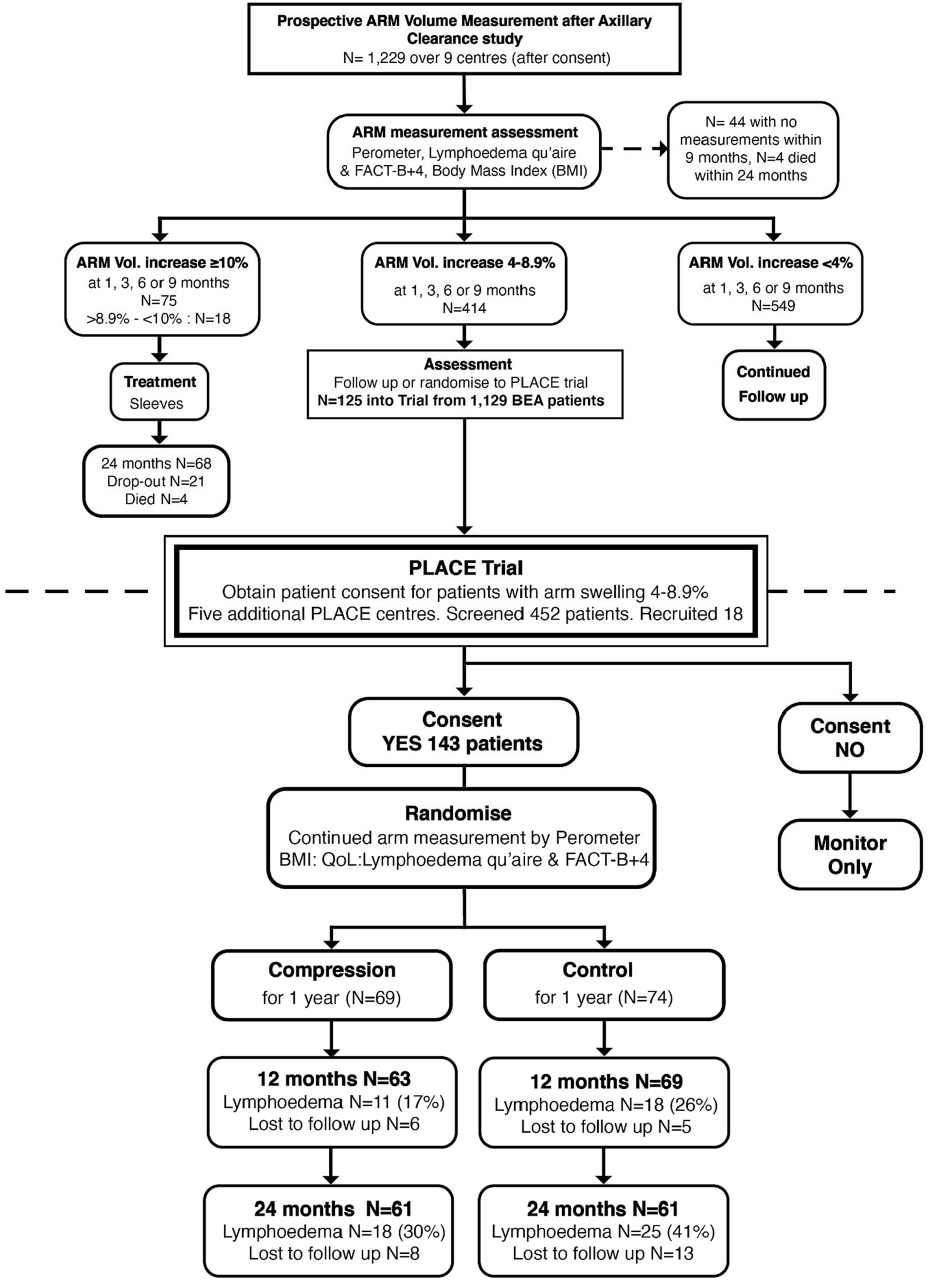
PLACE Trial Consort diagram. In the Flow diagram, the percentage Lymphoedema at each timepoint excludes the patients that dropped out, whereas in the main paper the percentage developing Lymphoedema is ITT so includes all patients

From these, participants developing RAVI between 4–9% within 9 months of surgery were recruited into this trial (PLACE) and subsequently randomised to compare intervention with a compression garment or standard management (Figure 1 Consort diagram).

Inclusion criteria: women aged 18–90 years with early breast cancer (no metastasis), who had undergone ANC.

Exclusion criteria: women with inoperable breast cancer (T4 category or distant metastasis), previous axillary radiotherapy or clearance prior to consent, past history of breast/chest wall radiotherapy prior to entering the trial (radiotherapy to chest wall or breast as part of treatment after clearance was allowed).

1. Intervention: application of graduated compression garments (Sigvaris:20‐‐24 mmHg) to the affected arm for 12 months (applied by trained lymphoedema practioners to ensure good fit) together with written advice on elevation, exercises and self‐massage.
2. Standard management: written advice on elevation, exercises and self‐massage.

Ethical approval for both the BEA and PLACE trial was granted by the ethics review board (REC 10/H1003/35).

All patients had a prestudy Body Mass Index (BMI) recorded and had BMI (weight) measurements repeated at each clinic visit to examine the potential relationship between high BMI and reduced compression sleeve efficiency.

### 2.1 Outcome measures

The primary outcome was proportion of participants developing lymphoedema (defined by RAVI > 10% from pre‐operative measurement) by 24‐months post‐randomisation in each arm as assessed by time to lymphoedema. Arm volume was measured at baseline, 1, 3, 6, 9 and 12 months then 6 monthly thereafter to 2 years, followed by annual measurements to 5 years after trial entry using a perometer.

### 2.2 Secondary outcomes

- Quality of Life (measured by Trial Outcome Index[TOI] and FACT‐B + 4) at 12, 18 & 24‐months post‐surgery,

The FACT‐B + 4 is a validated forty item cancer specific instrument which has 4 additional arm morbidity questions relevant to axillary surgery.^4^, ^7^ The TOI health score is derived from FACT‐B subscale scores. Quality of Life assessments: Quality of life questionnaires (TOI and FACT‐B + 4) and standard health utility measures were administered 6 monthly for 2 years then yearly to 5 years.

b Incidence of cellulitis,
c Incidence of moderate lymphoedema (RAVI > 20%) by 24‐months.
d Effect of BMI on lymphoedema incidence

Participants in the intervention group were fitted by a lymphoedema practitioner with a graduated compression garment (Sigvaris:CE, 20–24 mmHg round knit) which covered the whole arm from the wrist to the upper arm and was worn daily for 1 year after which it was discontinued. And 4 garments were provided to each participant to last for the year. All patients were reviewed 6 monthly. The patients in the control group, whose arm swelling increased to RAVI > 10% (lymphoedema) were considered to have failed control management and an appropriate compression sleeve provided as treatment by a lymphoedema therapist. Those in the intervention group whose RAVI increased to >10 were also recorded as having lymphoedema and their further 1 treatment delivered out of trial by lymphoedema nurses.^9^, ^10^, ^11^

The trial sought to change patient practice by empowering women to use arm sleeves to manage their own arm swelling, rather than consult lymphoedema nurses.

### 2.3 Statistical analysis

The sample size calculation was based on a two‐tailed two‐sample chi‐square test comparing the proportion of patients developing lymphoedema within 24‐months post‐randomisation, with a 1:1 treatment allocation ratio. Estimating that 45% of patients develop lymphoedema, to detect a 20% difference (i.e. 45% vs. 25%) in lymphoedema rate by 24‐months between the two treatment groups with 90% power and 5% significance level, requires 120 patients in each group but was increased to 135 per group to allow for dropouts.

Descriptive statistics are presented as Mean (SD) or Median (IQR), and as number (%) for continuous and categorical variables, respectively, unless otherwise stated. The analyses involved two‐tailed two‐sample tests with 5% significance level, performed on an intention‐to‐treat basis using statistical software R version 4.0.2. The primary analysis, incidence of lymphoedema within 24‐months post‐surgery, was assessed by chi‐square test. Quality of Life, measured by FACT‐B + 4 and TOI, was assessed by t‐test. Incidence of infection during follow‐up, and incidence of moderate/severe lymphoedema within 24‐months were assessed by Fisher's Exact test. Survival analysis was performed for time‐to‐lymphoedema, involving Kaplan–Meier curves and Cox regression. Additional exploratory analyses were performed for the primary outcome, to investigate the effect of BMI, and a per‐protocol analysis to take into account of protocol deviations.

## 3 RESULTS

From the 1229 participants screened in the BEA study, 414 developed a 4–9% RAVI within 9 months of ANC, of which 125 (Median Age 55 years) were recruited and randomised into the PLACE Trial (see Figure 1 CONSORT diagram). Due to slow recruitment, the PLACE study was opened to 5 more centres and a further 18 participants were recruited from a further 458 screened, thus 43 women (69 to the intervention group and 74 to the control group) entered the trial between 2011 and November 2015.

With continued low rate per month recruitment, the Independent Data Monitoring Committee (IDMC) advised that, even if recuitment of 200 patients was achieved, the outcome of the study was unlikely to alter and accordingly the trial was closed to new participants. Follow‐up of patients in the trial continued until at least 2 years after trial entry (August 2018). Median follow‐up was 43 months for patients in the intervention group, and 41 months in the control group.

Both groups were well matched for Body Mass Index (BMI), age, dominant arm, side of operation, smoking history, type of surgery and radiotherapy treatments. All patients underwent axillary clearance, 73 patients underwent mastectomy as surgical treatment and 70 patients breast conservation. Radiotherapy was given to Regional Nodes in 28 patients randomised to no sleeve and 27 to the sleeve application arm. Median follow‐up is 42 months (range 27.2–53.8) (see Table 1).

**TABLE 1:** Demographics of PLACE patients

		No sleeve (*n* = 74)	Sleeve (*n* = 69)
Age at randomisation		55.5 (33.5, 89.9)	55.8 (32.0, 86.9)
Body Mass Index (BMI) (pre op)		N = 72 27.8 (17.2, 45.3)	N = 67 28.7 (16.9, 60.9)
BMI (at PLACE entry)		26.9 (18.0, 47.1)	28.3 (16.9, 50.5)
Smoking history	Never	40	35
	Ex	20	25
	Current	5	9
Follow‐up (months from randomisation)		N = 72 41 (27.2, 53.8)	N = 69 43 (35, 53)
Tumour site	Upper Outer Quadrant	34	37
	Upper Inner quadrant	9	7
	Lower Outer Quad	9	2
	Lower Inner quadrant	6	2
	Central areolar	5	10
	Other	11	11
Side	Right:Left	29:36	23:41
Dominant hand	Right;Left	69;5	64:5
Grade	1	4 (5)	4 (6)
	2	32 (43)	31 (46)
	3	36 (49)	29 (43)
	Ungraded	2 (3)	3 (4)
Type of surgery	ANC	13	15
	WLocal Excision +ANC	23	17
	Mastectomy+ANC	36	34
	Other	2	3
Radiotherapy postop	Yes	64	60
Dose (cGy)		N = 64 4005 (3960, 5605)	N = 60 4005 (1068, 6010)
# Fractions		N = 64 15 (15, 25)	N = 60 15 (4, 30)
Site of radiotherapy	Breast	29	25
	Breast+SupraclavFossa	22	22
	Breast+Axilla	3	2
	Breast+SCF + Axilla	3	3
	Other	7	8
Adjuv chemotherapy	Yes	61 (82)	59 (86)
Number of nodes	Involved	2 (1–6.8)	3 (1–6)
	Removed	16 (13–22)	17 (11–22)
HER2	Negative	60 (81)	50 (72)
	Amplified or 3+	14 (18.9)	19 (27.5)
Receptor status	ER positive	55 (75)	58 (84)
	PR positive	32 (62)	29 (59)
RAVI Difference (at PLACE entry)		5.9 (4.1, 10.3)	5.9 (4, 8.5)
Time to lymphoedema (months)		4.5 (2.0–17.5)	9.9 (4.7–14.8)

*Note*: Median (Interquartile Range): Receptor status number (percentage). SCF radiotherapy to supraclavicular fossa nodal area.WLE (Wide Local excision). ANC (Axillary Node Clearance). NB not all patients underwent radiotherapy or adjuvant chemotherapy. Numbers (percentages undergoing treatment).

### 3.1 Primary outcome‐lymphoedema development

There was no difference in the proportion of patients who developed lymphoedema within 12 or 24 months in the No sleeve group (18 (26%) and 25 (41%) respectively) compared to the Sleeve group (11 (17%) and 18 (30% respectively:*p* = 0.32)). Twentyone percent (13) of patients randomised to No sleeve and 13% (8) in the Sleeve group were lost to follow‐up by 24‐months. Twentyeight (41%) and 20 (30%) of patients developed lymphoedema within 5‐years, in the No sleeve and Sleeve groups, respectively (*p* = 0.32).

**FIGURE 2:**
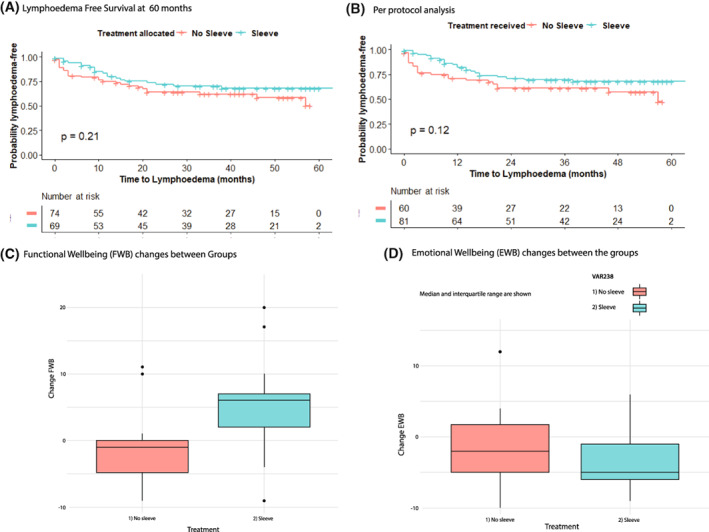
(A) Lymphoedema Free Survival at 60 months. (B) Per protocol analysis of Lymphoedema Free Survival. (C) Functional (FWB) and Emotional Well‐being (EWB) changes between Groups FACT‐B. Median and interquartile range are shown. (D) Emotional Well‐being (EWB) changes between the groups. Median and interquartile range are shown

For the subgroup who developed lymphoedema, Median (IQR) time to lymphoedema was 4.5 months (1, 17.5) for patients in the No sleeve arm, and 9.9 months (6, 15) for patients randomised to Sleeve. The Kaplan–Meier time‐to‐lymphoedema curves did not differ between groups in the Intention to treat and perprotocol analyses (*p* = 0.21 and *p* = 0.12: Figure 2A,B).

A total of 33 patients randomised to the ‘no sleeve’ group had a sleeve applied within 24 months;18 received the sleeve after measuring RAVI > 9%, 1 was lost to follow‐up and the remaining 13 patients had a sleeve applied before measuring RAVI > 9%.

In the per‐protocol analysis, 13 patients randomised to No sleeve were included as having received a Sleeve, with time‐to‐lymphoedema measured from the date the Sleeve was applied. Thirtyseven percent of the No sleeve patients (22/60) and 27% (22/81) of the per protocol sleeve patients developed lymphoedema within 24‐months. Similarly, 40% in the No sleeve group and 30% in the Sleeve group developed lymphoedema within 5‐years (Table 2).

**TABLE 2:** Incidence of Lymphoedema in per protocol analysis (RAVI ≥10% after Randomisation)

	No Sleeve	Sleeve	*p*‐value
	*n* = 59	*n* = 75	
Lymphoedema			
Within 2 years			
Yes	22 (37)	22 (27)	0.23
Within 5 years			
Yes	24 (40)	24 (30)	0.2

*Note*: In the per‐protocol analysis, 13 patients randomised to No sleeve were included as having received a Sleeve, with time‐to‐lymphoedema measured from the date the Sleeve was applied.

Body mass index (BMI) greater than 25 was present in 66% patients recruited to the trial. During the trial, the average change in BMI was a gain of 0.14 kg/m^2^ (IQR −4.43–3.87) with only 1 patient in each arm reducing their BMI to less than 30 and four in the no sleeve arm and 6 in the compression sleeve groups increasing their BMI over 30.BMI did not reduce from presurgery to randomisation despite advice on diet and exercise routinely provided to the patients in the study (Table 1).

### 3.2 Quality of life outcomes

Changes in Quality of Life (FACT‐B and TOI [Trial Outcome index]) from pre‐surgery to 12, 18 or 24 months post‐surgery did not differ between the two treatment groups (Table 3). At 12‐months, median (IQR) change in FACT‐B + 4 score was 0.5 points (−7, 9) in the No sleeve group, and 5 points (−5, 12) in the Sleeve group (*p* = 0.36) For TOI, median change was 3.5 points (−3, 10) in the No sleeve group and 4 points (−2, 13.5) in the Sleeve group (*p* = 0.33).

**TABLE 3:** Quality of Life assessment data

Variable	Change from pre‐surgery^a^, EMM (95% CI)				*p*‐value
	*n*	No sleeve	*n*	Sleeve	
FACT‐B at 12 months	43	2.60 (−1.66, 6.85)	46	3.44 (−0.67, 7.56)	0.78
TOI at 12 months	46	0.83 (−2.25, 3.91)	48	−0.11 (−3.12, 2.91)	0.67
ARM at 12 months	43	−3.67 (−4.93, −2.40)	43	−4.04 (−5.30, −2.77)	0.68
FACT‐B at 18 months	49	−3.25 (−7.41, 0.92)	47	1.88 (−2.37, 6.14)	0.091
TOI at 18 months	51	−3.09 (−6.18, 0.001)	49	−0.30 (−3.46, 2.85)	0.21
ARM at 18 months	42	−3.50 (−4.78, −2.21)	45	−3.33 (−4.57, −2.09)	0.85
FACT‐B at 24 months	44	−0.34 (−4.84, 4.16)	39	4.80 (0.01, 9.58)	0.12
TOI at 24 months	45	−0.43 (−3.72, 2.86)	41	1.14 (−2.30, 4.58)	0.51
ARM at 24 months	38	−1.73 (−3.21, −0.25)	35	−3.22 (−4.76, −1.68)	0.17

*Note*: The clinically important difference in Trial Outcome Index (TOI) is 5 points.

Abbreviation: EMM, estimated marginal mean.

a Calculated using an ANCOVA model with later time point as the dependent variable and pre‐surgery value and randomisation group as independent variable.

Changes in Functional Well Being scores in FACT‐B (No sleeve FWB ‐1 (−4.74, 0), versus Sleeve FWB: 6 (2, 7)) were significant (*p* = 0.007: Non‐parametric Wilcoxon/Mann Whitney U test: Figure 2C) Emotional Well Being changes (No sleeve −2 (−5, 1.75)) compared to Sleeve arm −5 (−6, −1) was not significant (*p* = 0.24).

Compression sleeves applied after development of Lymphoedema produced a short term improvement in QoL scores at 12 months (Table 4:FACT‐B *p* = 0.007:TOI *p* = 0.042). Which disappeared after 18 and 24 months.

**TABLE 4:** Effect of Sleeve application when Lymphoedema (RAVI > 10%) developed

Variable	Change from pre‐surgery^a^, EMM (95% CI)				*p*‐value
	*n*	No sleeve	*n*	Sleeve	
FACT‐B at 12 months	11	2.95 (−1.66, 7.56)	14	11.80 (7.71, 15.88)	0.007
TOI at 12 months	12	0.41 (−4.13, 4.95)	14	6.84 (2.63, 11.04)	0.042
ARM at 12 months	12	−3.17 (−5.26, −1.08)	13	−3.92 (−5.93, −1.91)	0.60
FACT‐B at 18 months	12	−2.96 (−10.82, 4.89)	14	4.69 (−2.58, 11.96)	0.15
TOI at 18 months	13	−3.82 (−9.84, 2.20)	14	2.18 (−3.62, 7.98)	0.15
ARM at 18 months	11	−2.44 (−4.32, −0.55)	14	−3.58 (−5.26, −1.91)	0.36
FACT‐B at 24 months	13	0.30 (−8.04, 8.63)	13	4.71 (−3.63, 13.04)	0.46
TOI at 24 months	13	−0.38 (−6.19, 5.43)	13	1.90 (−3.91, 7.71)	0.58
ARM at 24 months	11	−2.10 (−4.87, 0.68)	12	−4.41 (−7.07, −1.75)	0.22

*Note*: In patients in either arm the use of a sleeve once lymphoedema occurred improved QoL with increased FACT‐B and TOI scores at 12 but not 18 months. Conventionally a 5‐point increase in scores is considered clinically relevant.

Abbreviation: EMM, estimated marginal mean.

^a^ Calculated using an ANCOVA model with later time point as the dependent variable and pre‐surgery value and randomisation group as independent variables.

### 3.3 Incidence of cellulitis

Twelve (16%) patients in the no sleeve arm and 5 (7%) patients in the sleeve arm developed cellulitis of the affected arm during follow‐up (*p* = 0.12).

### 3.4 Incidence of moderate lymphoedema (RAVI > 20%) by 24‐months

No difference was found in the proportion of patients who developed moderate lymphoedema (RAVI > 20%) within 24‐months; 5% (4/74) in the No sleeve group, and 9% (6/69) in the Sleeve group (*p* = 0.66).

### 3.5 Effect of BMI on lymphoedema incidence

Body Mass Index (BMI) assessed as a continuous value at randomisation predicted lymphoedema at any time point HR 1.04 (CI 1.01–1.08; *p* = 0.02). 25/74 = 35% of patients in the No sleeve group, and 26/69 = 41% in the Sleeve group had obesity (BMI > 30) at randomisation. Of these patients with obesity, 13 (57%) in the No sleeve group and 10 (39%) in the Sleeve group developed lymphoedema by 24‐months. Of the patients with BMI < 30, 11 (26%) in the No sleeve group and 8 (21%) in the Sleeve group developed lymphoedema.

No difference was found in the two treatment groups (HR = 0.69 [0.39, 1.23], *p* = 0.21) for time to development of lymphoedema, and the difference remained non‐significant after adjusting for BMI at randomisation (HR = 0.61 [0.34–1.1], *p* = 0.1).

During the PLACE trial, the average change in BMI was a gain of 0.14 kg/m^2^ (IQR −4.43–3.87) with only 1 patient in each arm reducing their BMI to less than 30 and four in the no sleeve arm and 6 in the compression sleeve groups increasing their BMI over 30.

BMI did not reduce from presurgery to randomisation despite advice on diet and exercise routinely provided to the patients in the study (Table 1).

## 4 DISCUSSION

External Compression Garment application to the arm has been used as treatment for established arm LE for decades^1^, ^7^, ^9^, ^11^ despite the lack of evidence for the efficacy for compression therapy based on single centre studies.12

Following the^16^ claim that sleeve application in patients with early arm volume increases, prevented progression of arm swelling to LE,^16^ most international lymphoedema guidelines^9^, ^11^ have advised baseline arm volume or other measurements before surgery and intervention with compression sleeves if arm swelling (RAVI > 4%) occurs. This requires considerable time in patient outpatient visits and health economic costs.

The PLACE trial was designed to test the efficacy of such a strategy. Essentially, the reduction in lymphoedema with a compression garment should be considered as a percent of the control rate in a high risk population. Within 12 months using the sleeve, 17% of patients developed lymphoedema compared to 26% with best supportive care and 30% participants developed lymphoedema at 2 years in the intervention group. There was no evidence of benefit from surveillance and early application of a compression sleeve in subclinical LE in preventing clinical LE. Neither was a benefit of early intervention found either in terms of preventing lymphoedema progression to moderate LE or its infective complications.

It could be argued that early intervention may treat subclinical / mild clinical lymphoedema and therefore after 1 year in the intervention group we should see a difference compared with the control group which could disappear after the compression garment is discontinued. However, this was not the result found (see Figure 2A,B).

One limitation of the study is that we did not have an accurate record of adherence by participants to wearing the garment in the intervention group, despite the provision of diaries. Another limitation may be the different rate of loss to follow‐up by 24 months in the 2 groups (21% in the controls and 13% in the intervention group).

In this study, no benefit of early intervention was found either in terms of preventing lymphoedema progression to moderate LE or its infective complications. In the BEA study, high BMI was also associated with progression of lymphoedema after application of a compression sleeve.^7^ The poorer response to compression in obese patients is well recognised in lymphoedema clinics.

However, it should be recognised that although those who developed lymphoedema were followed up in the study, the treatment of their lymphoedema was provided by lymphoedema therapists outside the study and not standardised.

These findings will potentially apply to other cancers requiring axillary clearance (ie melanoma) causing lymphoedema following surgery.

Although PLACE patient recruitment was lower than planned, the similar rate of lymphoedema development in both trial arms indicates that there is no preventative effect of Compression Sleeves on Lymphoedema development compared to standard conservative management, particularly in overweight and obese patients. Notably, the small differences in lymphoedema rates was largely seen in the women with a normal BMI. Women with a normal BMI represent a minority of the cancer population. We had previously reported that in 271 nonrandomised BEA patients developed LE in 24% patients by 24 months despite sleeve application. Older Age, BMI > 30 and the number of metastatic nodes at axillary clearance predicted progression to Lymphoedema in those BEA patients.^7^ In the BEA study, high BMI was also associated with progression of lymphoedema after application of a compression sleeve.^7^

A small controlled trial randomised 45 women (23 in the compression group and 22 to the control) to light compression sleeves (15–21 mmHg) worn daily immediately after breast cancer surgery, yet two years later,^17^ 3 out of 20 patients from the compression group were still wearing their garments and 6 out of 21 from the control group had arm lymphoedema defined by an increased volume greater than 10% compared with preoperative values. There was no difference between the change in arm volume from preoperative values between the groups after two years, findings similar to our trial.^17^, ^18^ Stuiver et al found no evidence that class 2 compression stockings prevented lower limb lymphoedema in a trial of 85 patients undergoing groin dissection for cancer and they argued alternative prevention strategies were required.^19^

Following the^16^ claim that sleeve application in patients with early arm volume increases, prevent progression of arm swelling to LE,^16^ most international lymphoedema guidelines^9^, ^11^ have advised baseline arm volume or other measurements before surgery and intervention with compression sleeves if arm swelling (RAVI > 4%) occurs.

In the ALMANAC Trial of those patients developing 4–9% arm swelling by 6 months postsurgery, 30% improved spontaneously with standard management.^2^, ^4^ Many of these minor changes in arm swelling would not have been detected without preoperative measurements of both arms. In the BEA study 43% women mentioned arm swelling when asked at 6 months but only 10.5% had developed LE on measurement.^7^ Thus, 4–9% increase in arm swelling is usually clinically undetectable and asymptomatic. To screen such women thereby increasing patient anxiety while treating ephemeral arm changes is inappropriate and wasteful of health resources.

The lack of evidence of the effects of surveillance or compression sleeves on preventing LE raises doubts about the National Lymphoedema Network guideline recommendations.

Higher Lymphoedema risks for both overweight (OR 2) and obese (OR 3) patients after both sentinel^20^ and axillary node surgery^6^, ^7^, ^13^, ^21^ have been reported. We found BMI > 25 was associated with no treatment benefit from arm sleeve compression. Metaanalysis of Compression Sleeve Therapies in Lymphoedema^1^, ^12^ found any effect size was likely to be small, so it was not surprising we found little effect of the compression sleeve on preventing lymphoedema.

Weight Gain after Breast Cancer Surgery is common and due to the effects of chemotherapy and radiotherapy causing fatigue and steroid therapy during chemotherapy. Exercise and diet regimes reduce weight gain on adjuvant endocrine therapy but not chemotherapy^22^ Fluctuations in weight are reported to increase the risk of Lymphoedema.^23^

Weight loss has been found to reduce lymphoedema in a pilot study of overweight breast cancer survivors.^23^, ^24^

Two recent studies have shown upper ‐body exercise reduces lymphoedema flare‐ups and symptoms, possibly due to increased muscle function and vascular flow.^24^, ^25^ Additionally weightlifting has been found to reduce arm volume and lymphoedema when wearing a compression sleeve.^26^ The use of compression sleeves did not reduce progression of mild to moderate lymphoedema by five years after surgery nor did it reduce arm infections in the PLACE trial. However 80% of our patients underwent adjuvant chemotherapy and we have found that chemotherapy and particularly the corticosteroids prescribed during chemotherapy mitigate against any weight loss in the first year after surgery which may partly explain why chemotherapy increases the risk of lymphoedema.^7^

However the exercise interventions used in these studies above did not affect body weight^22^, ^23^, ^26^, ^27^ suggesting body weight loss, regimens, upper body exercises and compression sleeves may require to be tested in combination with exercise regimens as a management strategy for preventing lymphoedema.

The purpose of any screening intervention to prevent disease is to identify patients who will benefit from an intervention and prevent the disease being screened for subsequently developing. Early Intervention with compression sleeves did not prevent lymphoedema development.

The lack of preventative interventions suggests that screening for lymphoedema should not be recommended for all patients after axillary node clearance.

## AUTHOR CONTRIBUTIONS

**Emma Barrett:** Data curation (equal); formal analysis (equal); methodology (equal); software (equal); writing – review and editing (equal). **Chriss Todd:** Data curation (equal); formal analysis (equal); funding acquisition (equal); methodology (equal); project administration (equal); writing – original draft (equal); writing – review and editing (equal). **Donna Watterson:** Data curation (equal); project administration (equal); software (equal); writing – review and editing (equal). **Julie Morris:** Formal analysis (equal); methodology (equal); resources (equal); writing – original draft (equal). **Arnie Purushotham:** Project administration (equal); resources (equal); supervision (equal); writing – original draft (equal); writing – review and editing (equal). **Katie Riches:** Methodology (equal); project administration (equal); writing – original draft (equal). **Abigail Evans:** Project administration (equal); writing – original draft (equal); writing – review and editing (equal). **Anthony Skene:** Investigation (equal); project administration (equal); supervision (equal); writing – original draft (equal); writing – review and editing (equal). **Vaughan Keeley:** Conceptualization (equal); investigation (equal); methodology (equal); project administration (equal); supervision (equal); writing – original draft (equal); writing – review and editing (equal).

## FUNDING INFORMATION

The trial was funded by the UK National Institute for Health Research (NIHR) Programme Grant for Applied Research (RP‐PG‐0608‐10168), held by Professor Bundred (Chief Investigator).

Funding NIHR Programme grant. Protocol no: 2008/NJB/0503.dy. EudraCT no: 2008–001500‐22.

## CONFLICT OF INTEREST

There are no conflicts of interest.

## ETHICS APPROVAL AND CONSENT TO PARTICIPATE

The study was performed in accordance with the Declaration of Helsinki. The ethics was approved by the South Birmingham Research Ethics Committee. The participants all consented to take part in the study.

## CONSENT FOR PUBLICATION

All authors have provided their consent for publication.

## Data Availability

The data and material are all available through writing to Manchester CTU (formerly MAHSC‐CTU).
